# BYS10, a novel selective RET inhibitor, exhibits potent antitumor activity in preclinical models

**DOI:** 10.3389/fphar.2025.1670140

**Published:** 2025-10-16

**Authors:** Fei Qin, Yiman Chen, Jinhai Deng, Ruizhi Guo, Zhoufan Xie, Zhibo Luo, Lin Wang, Tianbai Huang, Jiaji Zhao, Jiansong Wang, Yingxia Bao

**Affiliations:** ^1^ Guangzhou Baiyunshan Pharmaceutical Holding Co., Ltd., Baiyunshan Pharmaceutical General Factory/Guangdong Province Key Laboratory for Core Technology of Chemical Raw Materials and Pharmaceutical Formulations, Guangzhou, China; ^2^ Kyinno Biotechnology Co., Ltd., Beijing, China; ^3^ School of Chemistry and Chemical Engineering, Guangdong Pharmaceutical University, Zhongshan, China

**Keywords:** BYS10, RET kinase inhibitor, solvent front mutations, selpercatinib resistance, molecular docking, antitumor activity

## Abstract

**Background:**

Aberrant alterations in the RET gene serve as oncogenic drivers in multiple cancers, making RET kinase inhibition a promising therapeutic strategy. However, acquired resistance limits the clinical efficacy of selective RET inhibitors.

**Methods:**

Enzymatic assays were used to measure the IC_50_ of BYS10 against wild-type RET and six mutants/fusions. The anti-RET activity of BYS10 was systematically evaluated through *in vitro* (cell proliferation inhibition assays) and *in vivo* (RET-altered xenograft models) experiments. RET phosphorylation inhibition by BYS10 was confirmed via Western blot, and optimized binding for RET G810R/S potent inhibition was verified by molecular docking.

**Results:**

In enzymatic assays, BYS10 showed low nanomolar potency against wild type RET and six clinically relevant RET mutations/fusions, including RET G810R/S (IC_50_ 0.01–3.47 nM) and RET V804M/L (IC_50_ 2.18–2.65 nM). BYS10 also displayed significant anti-proliferative activity across a panel of RET-altered cell lines, including the inhibition of Ba/F3-KIF5B-RET-G810R/S (IC_50_ 25.94–240.60 nM) and Ba/F3-KIF5B-RET-V804M/L (IC_50_ 13.38–46.09 nM). Supported by favorable pharmacokinetics, BYS10 achieved robust anti-tumor efficacy in diverse RET-driven xenograft models. In Ba/F3-KIF5B-RET xenograft model, BYS10 at 3 mg/kg achieved a TGI% of 78.45%, versus 57.06% for Selpercatinib (P < 0.001). In Ba/F3-KIF5B-RET-V804L xenograft model, BYS10 at 3 mg/kg achieved a TGI% of 94.67%, versus 79.48% for Selpercatinib (P < 0.05). In Ba/F3-KIF5B-RET G810R xenograft model, BYS10 at 10 mg/kg achieved a TGI% of 65.96%, versus 35.37% for Selpercatinib (P < 0.001). In Ba/F3-KIF5B-RET G810S xenograft model, BYS10 at 10 mg/kg achieved a TGI% of 112.59%, versus 82.15% for Selpercatinib (P < 0.001). Western blot analysis confirmed potent suppression of RET phosphorylation by BYS10. Molecular docking analysis confirmed that BYS10 achieves potent inhibition of RET G810R/S proteins through an optimized binding mode.

**Conclusion:**

Collectively, BYS10 represents a novel, highly selective RET inhibitor with superior *in vitro* and *in vivo* activity against multiple RET alterations compared to Selpercatinib. Its recent Investigational New Drug (IND) approvals from the FDA and NMPA underscore its therapeutic potential for RET-driven malignancies.

## 1 Introduction

The rearranged during transfection (RET) proto-oncogene, first identified by Takahashi et al. in 1985 (Takahashi et al., 1985), encodes a transmembrane glycoprotein receptor tyrosine kinase. This kinase plays critical roles in embryonic development of renal, neural, and neuroendocrine tissues (Mullally et al., 2025). Aberrant RET signaling drives multiple malignancies including non-small cell lung cancer (NSCLC), medullary thyroid carcinoma (MTC), papillary thyroid carcinoma, pancreatic cancer, and prostate cancer (Ahn et al., 2020; Cascetta et al., 2021). RET activation occurs through mutations, gene fusions, and overexpression (Thein et al., 2021), with KIF5B (70%–90%) and CCDC6 (10%–25%) constituting the predominant fusion partners in RET-rearranged NSCLC (Aldea et al., 2023; Hoe and Solomon, 2025). These alterations enable ligand-independent constitutive activation of RET, triggering autophosphorylation and persistent stimulation of downstream pathways—including Ras/MAPK, PI3K/AKT, and JNK—that promote cellular proliferation, malignant transformation, and tumorigenesis (Subbiah et al., 2020; Qiu et al., 2024). Basket trial successes have validated RET as a promising therapeutic target (Subbiah et al., 2024), suggesting its potential as a new diamond gene in the future (Gou et al., 2022).

First-generation RET kinase inhibitors are multikinase inhibitors (MKIs), exemplified by sorafenib and sunitinib. In 2020, the U.S. Food and Drug Administration (FDA) approved second-generation selective RET inhibitors, selpercatinib and pralsetinib. These agents significantly enhance therapeutic efficacy and safety profiles through precise targeting of the RET kinase domain (Gainor et al., 2021; Drilon et al., 2023), establishing small-molecule RET-specific inhibitors as standard-of-care for advanced RET-driven malignancies. Despite their promising efficacy, acquired resistance inevitably emerges, frequently involving solvent-front mutations that drive disease progression (Ali et al., 2022; Clark et al., 2023). To address this challenge, third-generation selective RET inhibitors—including TPX-0046 (Repetto et al., 2022), BOS172738 (Chen et al., 2024), TAS0953 (Miyazaki et al., 2023), and LOXO-260 (Lin and Gainor, 2023)—are currently under clinical development. Ongoing research remains critical to overcome resistance mechanisms associated with RET alterations.

BYS10, a novel highly selective third-generation RET inhibitor, is currently undergoing Phase I/II clinical trials (Wang et al., 2024). We demonstrated that BYS10 potently inhibits solvent-front (G810R/S), gatekeeper (V804L/M), and catalytic loop (M918T) mutations in both biochemical and cellular assays. Furthermore, BYS10 exhibited significant efficacy against selpercatinib-resistant, RET-G810R/S-driven fusion tumors in xenograft models. These findings suggest substantial clinical potential for developing novel RET inhibitors to treat patients resistant to existing therapies.

## 2 Materials and methods

### 2.1 Enzyme activity assays

The kinase activity was detected using the HotSpot testing platform of Rection Biology Corporation (Anastassiadis et al., 2011). Prepare the reaction buffer freshly, add the kinase to be tested, and mix gently. BYS10 and Selpercatinib (provided by Shanghai Wuxi AppTec New Drug Development Co., Ltd.) were transferred into the reaction solution using the acoustic liquid handling non-contact technology (Echo550). ^33^P-ATP was added to the reaction system and incubated at room temperature for 120 min, followed by spotting of the reactions onto P81 ion exchange filter paper. Unbound phosphate was removed by extensive washing of filters in 0.75% phosphoric acid and the radioactively phosphorylated substrate on the filter membrane was detected. The Prism software (GraphPad) was used for curve fitting and calculation of the IC_50_ value.

### 2.2 Molecular docking

The molecular docking of wild-type Ret kinase with BYS10 was performed using the separated receptor from the co-crystal structure (PDB ID: 7du8). For the molecular docking of Ret-G810S and Ret-G810R mutant kinases with BYS10, homology modeling was applied. Molecular docking was performed using AutoDock Vina, visualization was conducted with PyMOL, and protein-ligand interaction analysis was carried out using the PLIP tool.

To prepare the receptors, the co-crystal ligands, waters and irrelevant heteroatoms were removed from the 7du8 complex to yield the pure receptor, which was added hydrogen and Gasteiger charge, and saved as PDBQT file using the scripts provided by MGLTooLs. The ligands were casually optimized with ChemDraw 3D, added gasteiger charge, and set torsion roots. The coordinate of docking grid center was determined by calculating the geometric center of all atoms in the co-crystallized ligand with a python script. The size of the grid box was set to be 40 Å. The current docking simulation was performed rigidly, with no flexible residues defined, as preliminary tests indicated that rigid docking alone could yield results consistent with experimental data. The redocking RMSD vs. the co-crystal ligand of 7du8 was 0.379 Å. The binding free energies were calculated using vina score.

### 2.3 Cell culture

Ba/F3-KIF5B-RET, Ba/F3-CCDC6-RET, Ba/F3-KIF5B-RET-V804L, Ba/F3-KIF5B-RET-V804M, Ba/F3-KIF5B-RET-G810R, and Ba/F3-KIF5B-RET-G810S cells were provided by Kyinno Biotechnology Co., Ltd. The cells were cultured in RPMI 1640 (Biological Industries, Israel) supplemented with 10% fetal bovine serum (Biological Industries, Israel) and 1% Penicillin/Streptomycin solution (Corning, United States). They were incubated at 37 °C in a humidified atmosphere containing 5% CO_2_, and cell proliferation was evaluated using the Celltiter-Glo luminescent cell viability assay.

### 2.4 *In Vivo* xenograft model

Animal experiments were approved by the Institutional Animal Care and Use Committee of Kangyuan Bochuang Biotechnology (Beijing) Co., Ltd. (No.KY-AUP-W250418). B-NDG and NPSG mice were purchased from Beijing Biocytogen Co., Ltd. and Shanghai Jihui Laboratory Animal Breeding Co., Ltd., respectively. The right flanks of B-NDG and NPSG mice were subcutaneously injected with 1 × 10^6^ Ba/F3-KIF5B-RET and Ba/F3-KIF5B-RET-V804L cells, respectively. NOD SCID mice were purchased from Beijing GemPharmatech Co., Ltd. The right flanks of NOD SCID mice were subcutaneously injected with 1 × 10^6^ Ba/F3-KIF5B-RET-G810R/G810S cells, respectively. When the average tumor volume reached 80–120 mm^3^, tumor-bearing mice were randomly divided into five groups and treated with compounds or vehicles (n = 8). Compounds were orally administered, twice daily for 11-19 consecutive days. Tumor volumes were calculated as L × W × W/2 (where L and W represent the length and width of the tumor, respectively). At the experiment terminal, mice were euthanized by CO_2_ inhalation at a displacement rate of 30%–70% of the chamber volume per minute.

### 2.5 Pharmacokinetic study

After successfully establishing the above-mentioned Ba/F3-KIF5B-RET mouse model, the mice in the BYS10 (1, 3, 10 mg/kg) groups underwent a PK study on the 12th day of drug administration. Blood samples were collected at the time points of 15 min, 30 min, 1 h, 2 h, 4 h, 8 h, 12 h and 24 h after a single drug administration, and the compound concentration was determined using LC-MS/MS.

### 2.6 Western blot

Ba/F3-KIF5B-RET-V804L cell suspension was inoculated into NPSG immunodeficient mice. When the average tumor volume was around 500 mm^3^, 15 mice were selected and randomly divided into 5 groups, with 3 mice in each group. The animals were humanely sacrificed 1 h after drug administration, and the tumor tissues were collected and quickly frozen in liquid nitrogen for subsequent testing. The frozen tumor tissues were lysed with protein lysis buffer. The centrifuged tissue homogenate was diluted 5 times for protein concentration quantification. After denaturation at 100 °C, electrophoresis was carried out using a PAGE gel, and then the proteins were transferred onto a PVDF membrane. After blocking at room temperature for 1 h, the membrane was incubated overnight at 4 °C with diluted primary antibodies against RET, pRET and β-actin respectively. Then it was incubated with diluted rabbit secondary antibody at room temperature for 90 min. Finally, it was developed with ECL and imaged using a gel imaging instrument.

### 2.7 Statistical methods

The results are presented as mean ± standard deviation (SD). Data were analyzed using SPSS 24.0 software. Two groups were compared using a two-tailed Student’s t-test. Comparisons between multiple groups were performed using a one-way analysis of variance (ANOVA) followed by multiple comparisons Tukey test. *P* < 0.05 was defined statistically significant.

## 3 Results

### 3.1 Binding selectivity

**FIGURE 1:**
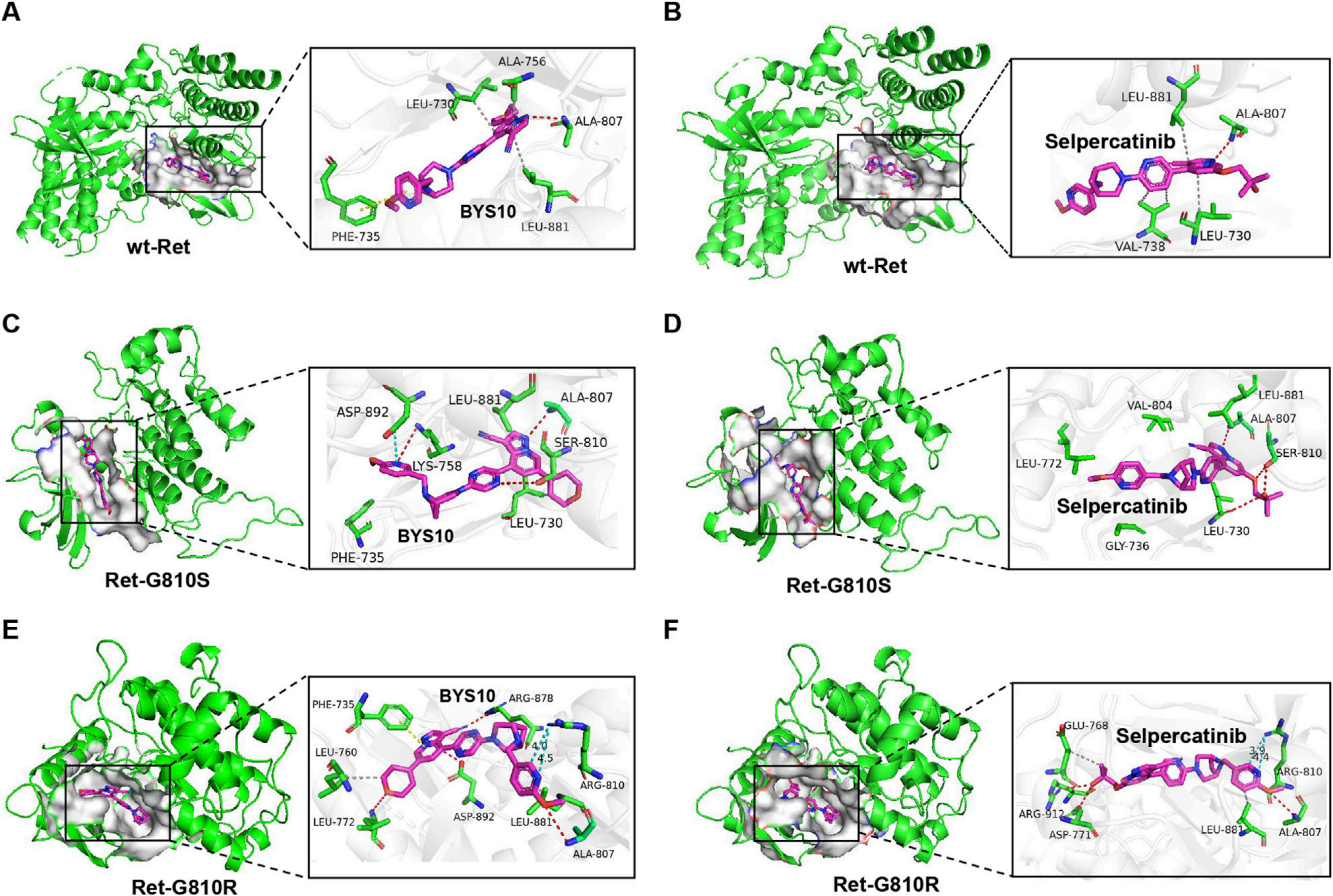
Molecular docking results of **(A)** wild-type Ret kinase with BYS10; **(B)** wild-type Ret kinase with Selpercatinib; **(C)** Ret-G810S with BYS10; **(D)** Ret-G810S with Selpercatinib; **(E)** Ret-G810R with BYS10; **(F)** Ret-G810R with Selpercatinib.

BYS10 was developed through a structure-based drug design approach. As shown in Figure 1, BYS10 binds to the ATP-binding pocket in both wild-type and mutant (G810S/G810R) Ret kinases. In the wild-type Ret kinase (Figures 1A,B), the imidazopyridine heterocycle of BYS10 forms a hydrogen bond with the key hinge residue Ala807, while also engaging in hydrophobic interactions with the hydrophobic pocket formed by Leu730 and Leu881. Additionally, the dihydropyran side chain of BYS10 participates in a π-π stacking interaction with the Phe735 residue. The binding affinity and interaction patterns of BYS10 and Selpercatinib with wild-type RET kinase are similar.

In the G810S mutant (Figures 1C,D), the pyridine side chain of BYS10 forms hydrogen bonds and a salt bridge with Lys758 and Asp892 of the G810S mutant, while the tertiary alcohol side chain of Selpercatinib establishes a hydrogen bond with Leu730 of the G810S mutant. As for BYS10, its highly planar dihydropyran side chain and the small dihedral angle with the imidazopyridine ring enable deeper penetration into the hydrophobic pocket, resulting in stronger binding to the G810S mutant compared to Selpercatinib.

In the G810R mutant, the binding conformations of the ligands differ significantly from those observed in both the wild-type and G810S mutant. The bulky Arg810 mutation increases steric hindrance in the hinge region, preventing the steric imidazopyridine ring from approaching the Ala807 residue. Instead, the pyridine side chain forms a hydrogen bond with Ala807. The large tertiary alcohol side chain of Selpercatinib is hindered by Arg810 at the hinge region, making it difficult to penetrate deeply into the hydrophobic cavity (Figure 1F). In contrast, the dihydropyran side chain of BYS10 adopts a quasi-planar cyclic structure, and enters the hydrophobic pocket formed by Leu760 and Leu772 with a small dihedral angle relative to the pyrazolopyridine ring, thereby avoiding steric clashes with the bulky Arg810 (Figure 1E). The cyano group in BYS10 forms a hydrogen bond with Arg878, while the pyrazolopyridine scaffold not only establishes a salt bridge with Asp892 but also engages in π-π stacking with Phe735. Additionally, the distance between Arg810 and the pyridine side chain of BYS10 is slightly greater than that with Selpercatinib, potentially resulting in weaker electrostatic repulsion between the basic mutant residue and BYS10’s pyridine side chain compared to Selpercatinib. In summary, BYS10 is less affected by the Arg810 mutation in terms of binding to the Ret-G810R mutant compared to Selpercatinib. As shown in Table 1, the optimal binding conformation of BYS10 exhibits a binding free energy of −11.65 kcal/mol with RET, superior to that of Selpercatinib (−10.37 kcal/mol).

**TABLE 1:** *In vitro* kinase activity assays for different RET kinase mutation or rearrangement forms.

Kinase	IC_50_ (nM, mean ± SD)	
	BYS10	Selpercatinib
RET	0.46 ± 0.06	0.55 ± 0.09
RET (M918T)	2.63 ± 0.66	3.87 ± 0.94
RET (V804L)	2.18 ± 0.60	3.41 ± 0.87
RET (V804M)	2.65 ± 0.71	3.97 ± 1.05
RET (G810S)	0.01 ± 0.002	2.72 ± 0.71
RET (G810R)	3.47 ± 0.98	88.80 ± 24.00
RET-CCDC6 (PTC1)	0.57 ± 0.17	0.81 ± 0.23

### 3.2 Kinase selectivity

We evaluated the inhibitory activity of BYS10 against RET wild-type and clinically relevant mutants: RET-M918T, RET-V804L, RET-V804M, RET-G810S, RET-G810R, and the RET-CCDC6 (PTC1) fusion kinase. BYS10 potently inhibited six major oncogenic RET variants at nanomolar concentrations, demonstrating significantly enhanced activity over selpercatinib (Table 1). Notably, BYS10 exhibited potent inhibition against both RET-G810S (IC_50_ = 0.01–3.47 nM) and RET-G810R (IC_50_ = 0.02–1.89 nM), with 25- to 270-fold greater potency than selpercatinib across these mutants.

**FIGURE 2:**
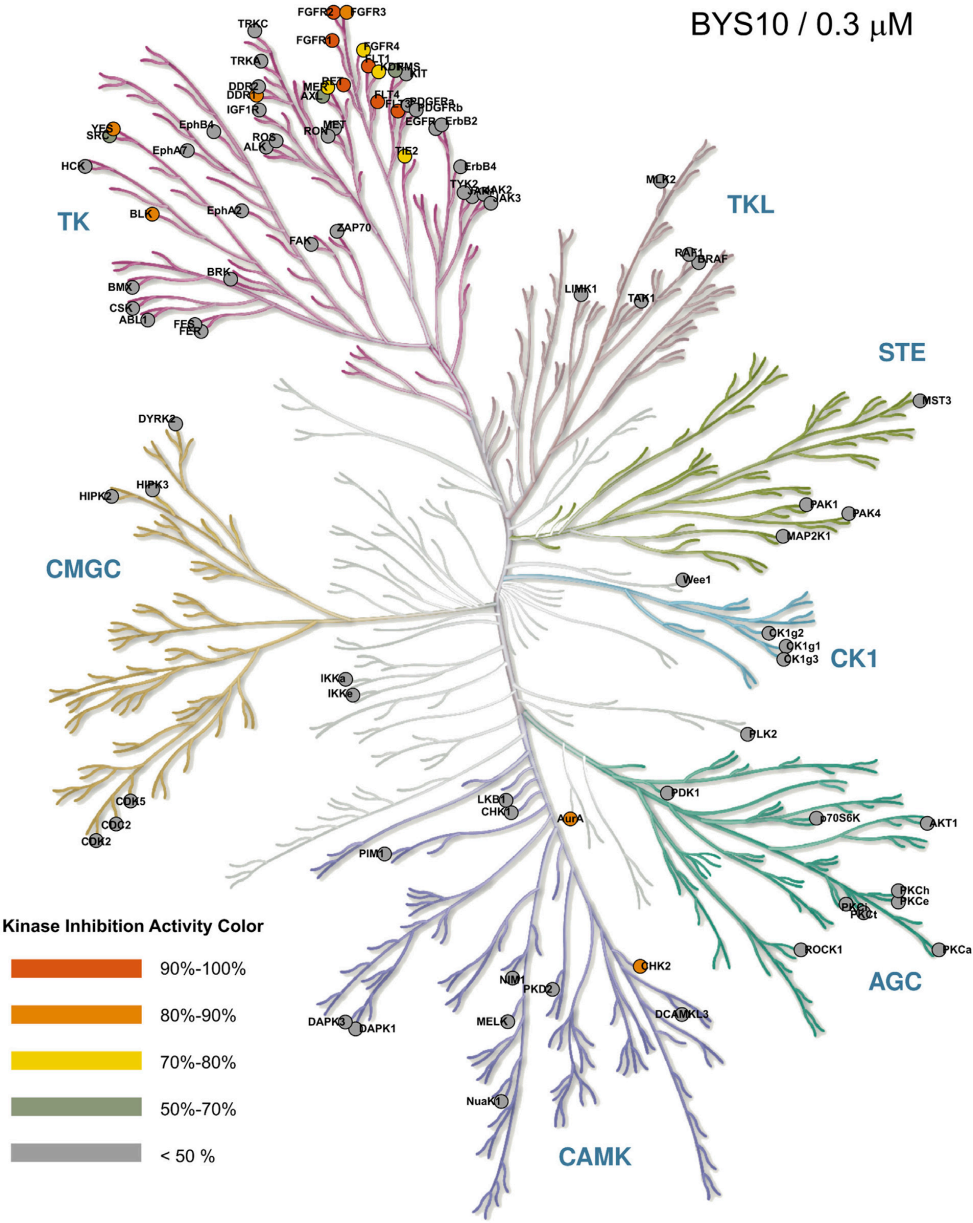
Selectivity profile of BYS10 against 111 kinases at a concentration of 0.3 μM. The inhibition rates (IC_50_) were determined using the Reaction Biology Corporation.

To assess the kinase selectivity profile of BYS10, we screened its inhibitory activity at 0.3 μM against a panel of 111 kinases. As shown in Figure 2, BYS10 exhibited >85% inhibition against 10 kinases: BLK, c-KIT (D816H), c-SRC, FGFR1, FGFR2, FLT1, FLT3, FLT3 (D835Y), FLT4, and KDR. Concentration-response studies determined IC_50_ values for these hits. Notably, BYS10 demonstrated >10-fold selectivity for RET over these off-target kinases.

### 3.3 *In vitro* anti-proliferative activity

Given that KIF5B and CCDC6 represent the most frequent RET fusion partners, we evaluated the anti-proliferative activity of BYS10 in cell lines expressing wild-type fusions (KIF5B-RET and CCDC6-RET) and resistance-associated mutants (KIF5B-RET-V804M, KIF5B-RET-V804L, KIF5B-RET-G810S, and KIF5B-RET-G810R) (Table 2). BYS10 demonstrated significantly enhanced potency over selpercatinib. Notably, in Ba/F3 cells expressing solvent-front mutant fusions (KIF5B-RET-G810S and KIF5B-RET-G810R), BYS10 exhibited 3–5-fold greater potency than selpercatinib. These results indicate BYS10 overcomes resistance mediated by RET solvent-front mutations in selpercatinib-resistant models.

**TABLE 2:** Anti-proliferative Activity of BYS10 against RET and mutants in established Ba/F3 cells.

Cell line	IC_50_ (nM, mean ± SD)	
	BYS10	Selpercatinib
Ba/F3-KIF5B-RET	5.97 ± 0.64	11.48 ± 4.31
Ba/F3-KIF5B-RET-V804L	13.38 ± 1.28	16.20 ± 3.22
Ba/F3-KIF5B-RET-V804M	46.09 ± 8.11	65.32 ± 20.69
Ba/F3-KIF5B-RET-G810R	240.60 ± 44.63	734.23 ± 56.07
Ba/F3-KIF5B-RET-G810S	25.94 ± 1.73	124.69 ± 19.74
Ba/F3-CCDC6-RET	55.86 ± 21.40	70.28 ± 0.91

**FIGURE 3:**
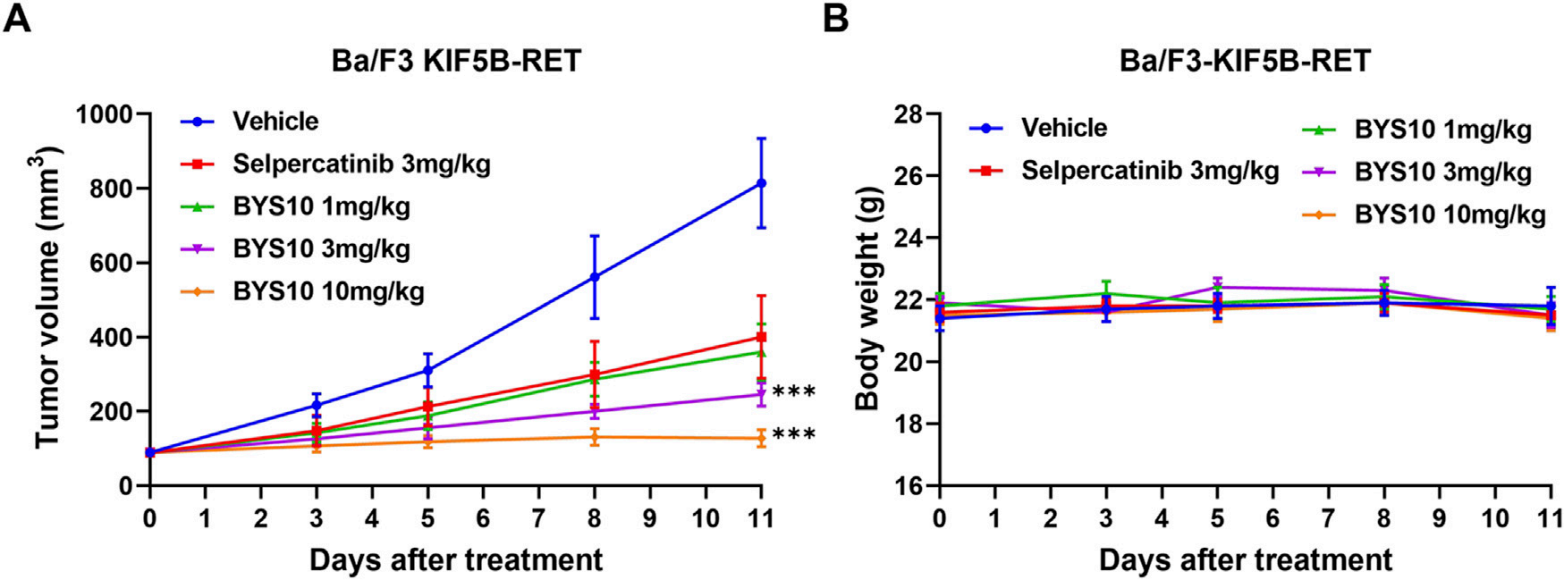
Efficacy of BYS10 inhibits tumorigenesis in B-NDG mice bearing Ba/F3-KIF5B-RET tumor. The change of tumor volume **(A)** and body weight **(B)** of mice were presented in graphs. (n = 8, p.o. BID. Vehicle is 5%DMSO+10%solutol+85%water. The tumor volume and body weight of mice were measured at days 0, 3, 5, 8 and 11 after treatment.) Data are mean ± SD. ***P < 0.001 versus Selpercatinib.

Efficacy and preliminary PK Study in Ba/F3-KIF5B-RET Xenograft Mouse Model Given BYS10’s potent *in vitro* anti-proliferative activity (Table 2), we evaluated its *in vivo* efficacy using a Ba/F3-KIF5B-RET xenograft mouse model. As shown in Figure 3A, BYS10 significantly inhibited tumor growth in a dose-dependent manner, with the tumor growth inhibition (TGI) values of 62.68%, 78.45%, and 94.64% at doses of 1, 3, and 10 mg/kg, respectively. While the TGI value of Selpercatinib (3 mg/kg) was 57.06%. The treatment schedules were well tolerable without visible loss of animal body weight (Figure 3B). A concomitant pharmacokinetic study on Day 12 revealed dose-proportional increases in plasma exposure (Table 3). The results showed that BYS10 achieved effective plasma exposure in mice, with C_max_ and AUC_0-last_ values increasing linearly with the dose, demonstrating good pharmacokinetic properties.

**TABLE 3:** Pharmacokinetic properties of BYS10 in mice.

Group	1 mg/kg	3 mg/kg	10 mg/kg
C_max_ (ng/mL)	409	1220	3770
T_max_ (h)	1.00	0.500	1.00
T_1/2_ (h)	4.15	4.04	2.60
AUC_0-last_ (ng.h/mL)	4090	12600	35900
AUC_0-inf_ (ng.h/mL)	4170	12900	36000

### 3.4 Efficacy and PD study in Ba/F3-KIF5B-RET-V804L xenograft mouse model

**FIGURE 4:**
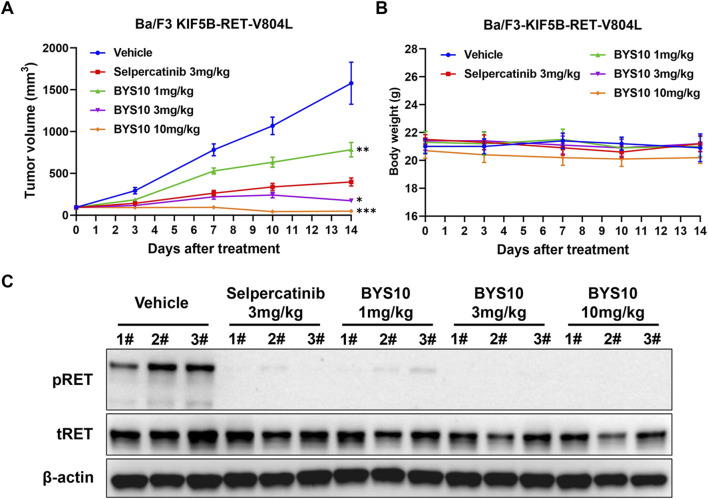
Efficacy of BYS10 inhibits tumorigenesis in NPSG mice bearing Ba/F3-KIF5B-RET-V804L tumor. The change of tumor volume **(A)** and body weight **(B)** of mice were presented in graphs; **(C)** RET phosphorylation was inhibited by BYS10 in tumors. (n = 8, p.o. BID. Vehicle is 5%DMSO+10%solutol+85%water. The tumor volume and body weight of mice were measured at days 0, 3, 7, 10 and 14 after treatment.) Data are mean ± SD. *P < 0.05, **P < 0.01, ***P < 0.001 versus Selpercatinib.

We next evaluated BYS10 in a Ba/F3-KIF5B-RET-V804L xenograft model. BYS10 significantly inhibited tumor growth in a dose-dependent manner, with TGI values of 53.55%, 94.67%, and 103.02% at doses of 1, 3, and 10 mg/kg, respectively (Figure 4A). While the TGI value of Selpercatinib (3 mg/kg) was 79.48%. No significant weight loss or obvious clinical abnormalities were observed in mice of BYS10 group (Figure 4B). Pharmacodynamic analysis demonstrated dose-dependent inhibition of RET phosphorylation in tumors harvested 1 h post-administration. Western blot analysis revealed near-complete phospho-RET suppression at 1 mg/kg and complete inhibition at ≥3 mg/kg (Figure 4C), confirming potent target inhibition.

### 3.5 Efficacy study in Ba/F3-KIF5B-RET-G810R/S xenograft mouse model

**FIGURE 5:**
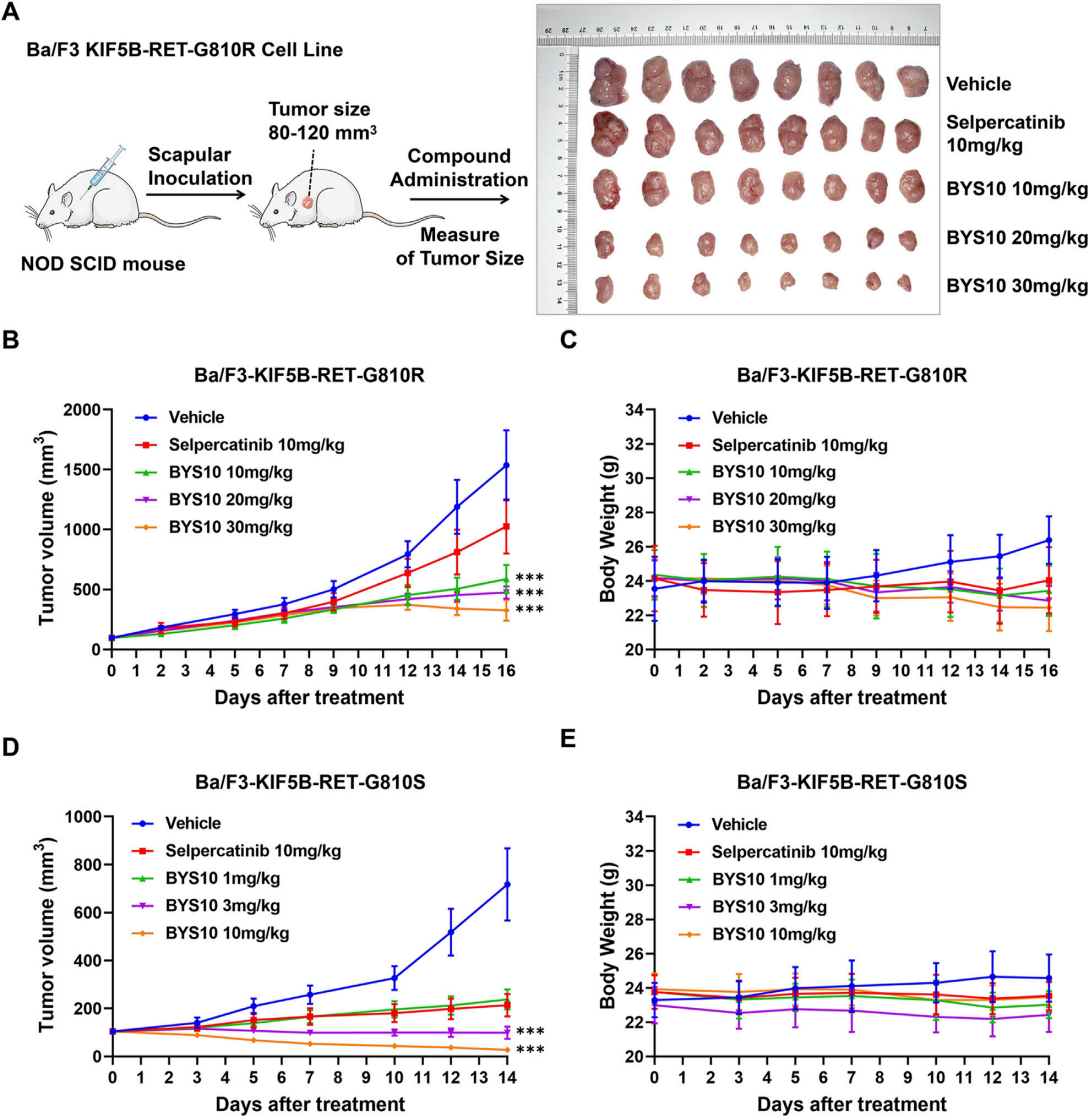
Efficacy of BYS10 inhibits tumorigenesis in NOD SCID mice bearing Ba/F3-KIF5B-RET-G810R/S tumor. **(A)** The administration plan in BaF3/KIF5B-RET-G810R xenograft model and images of dissected tumor tissues. **(B)** Ba/F3-KIF5B-RET-G810R Tumor volume changes during experiment. **(C)** Body weight changes during the experiment (n = 8. p.o. BID. Vehicle is 5%DMSO+10%solutol+85%water. The tumor volume and body weight of mice were measured at days 0, 2, 5, 7, 9, 12, 14 and 16 after treatment.) **(D)** Ba/F3-KIF5B-RET-G810S Tumor volume changes during experiment. **(E)** Body weight changes during the experiment (n = 8. p.o. BID. Vehicle is 5%DMSO+10%solutol+85%water. The tumor volume and body weight of mice were measured at days 0, 3, 5, 7, 10, 12, and 14 after treatment.) Data are mean ± SD. ***P < 0.001 versus Selpercatinib.

The *in vivo* antitumor efficacy of BYS10 was next evaluated in NOD SCID mice bearing the Ba/F3-KIF5B-RET-G810R/S xenograft model. In the BaF3-KIF5B-RET-G810R cell-derived xenograft model, BYS10 was administered at doses of 1, 3, and 10 mg/kg on days 0–7; to enhance efficacy, the dose was escalated to 20 mg/kg (for the initial 1 mg/kg group), 30 mg/kg (for the initial 3 mg/kg group), and maintained at 10 mg/kg (for the original 10 mg/kg group) during days 8–16. The TGI of BYS10 (10, 20, and 30 mg/kg) were 65.96%, 73.71%, and 83.98%, respectively. While the TGI of Selpercatinib (10 mg/kg) was 35.37% (Figure 5B). These results demonstrate BYS10’s superior efficacy against G810R-mutant tumors compared to selpercatinib (Figure 5A).

We also found that BYS10 significantly inhibited the growth of tumors in the Ba/F3-KIF5B-RET-G810S model. The TGI of BYS10 (1, 3, and 10 mg/kg) were 78.29%, 100.99%, and 112.59%, respectively, almost completely inhibiting tumor growth (Figure 5D). The TGI of the Selpercatinib (10 mg/kg) was 82.15%. It can be seen that at the same dosage, BYS10 has a better inhibitory effect on G810S mutant RET xenograft tumors than Selpercatinib. During the experimental period, no significant weight loss or obvious clinical abnormalities were observed in mice of BYS10 group (Figures 5C,E).

## 4 Discussion

Oncogenic activation of RET kinase through fusion or mutation drives tumor cell proliferation and survival, establishing it as both a diagnostic marker and therapeutic target across multiple cancers. RET fusions occur in ∼10% of papillary thyroid carcinomas (PTC), 1%–2% of non-small cell lung cancers (NSCLC), and rarely in other malignancies (Addeo et al., 2023). CCDC6-RET and KIF5B-RET represent the predominant fusion isoforms in PTC and NSCLC, respectively (Li et al., 2019; Parimi et al., 2023). Catalytic domain mutation M918T and gatekeeper mutations V804L/M frequently occur in multiple endocrine neoplasia type 2 (MEN2) and medullary thyroid carcinoma (Li et al., 2021; Salvatore et al., 2021). While first-generation inhibitors (selpercatinib, pralsetinib) improve outcomes in RET-altered cancers, solvent-front mutations (notably G810R/S/C) confer therapeutic resistance (Qiao et al., 2024). G810R induces the most profound resistance, rendering selpercatinib ineffective (Lin et al., 2020), with pralsetinib demonstrating cross-resistance to these variants (Subbiah et al., 2021). Consequently, next-generation RET inhibitors capable of overcoming acquired resistance while maintaining high selectivity represent an urgent clinical need.

Molecular docking analysis confirmed that BYS10 achieves potent inhibition of RET G810R/S proteins through an optimized binding mode. Biochemically, We found that BYS10 exhibited potent inhibition against RET kinase and six mutants, outperforming Selpercatinib. Notably, BYS10 exhibited superior anti-proliferative activity compared to Selpercatinib against KIF5B-RET-G810R/S mutation. It also suppressed Ba/F3-CCDC6-RET, Ba/F3-KIF5B-RET, Ba/F3-KIF5B-V804 L/M, Ba/F3-KIF5B-G810R/S, outperforming Selpercatinib. *In vivo* antitumor experiments showed that BYS10 significantly inhibited tumor growth in a dose-dependent manner. In Ba/F3-KIF5B-RET xenograft model, BYS10 at 3 mg/kg achieved a TGI of 78.45%, versus 57.06% for Selpercatinib. To verify BYS10’s ability to overcome RET gatekeeper mutations *in vivo*, we assessed its antitumor activity in the Ba/F3-KIF5B-RET-V804L xenograft model. In this model, BYS10 at 3 mg/kg achieved a TGI of 94.67%, versus 79.48% for Selpercatinib. To verify BYS10’s ability to overcome RET solvent front mutations *in vivo*, we assessed its antitumor activity in the Ba/F3-KIF5B-RET-G810R/S xenograft model. In Ba/F3-KIF5B-RET-G810R xenograft model, BYS10 at 10 mg/kg achieved a TGI of 65.96%, versus 35.37% for Selpercatinib. In Ba/F3-KIF5B-RET-G810S xenograft model, BYS10 (1 mg/kg), 10 times less than the dose of Selpercatinib (10 mg/kg), obviously inhibited tumor growth, with TGI value of 78.29%. BYS10 (3 mg/kg) completely inhibited tumor growth, with TGI value of 100.99%. As can be seen, BYS10 demonstrated superior antitumor activity against both RET gatekeeper mutations and solvent front mutations. The 2D interaction map of BYS10 and Selpercatinib with RET-G810R and the overlap of BYS10 and Selpercatinib in RET-G810R was provided in SI.

Studies have shown that certain next-generation RET inhibitors inhibit RET solvent front mutations preclinically but they lack inhibitory activity against RET gatekeeper mutations (Drilon et al., 2019). However, selective inhibition of both mutations may be important for patients. The development of a next-generation selective RET kinase inhibitor capable of inhibiting RET solvent front and gatekeeper mutations simultaneously holds significant clinical value (Solomon et al., 2020). BYS10 is precisely such a novel RET inhibitor capable of simultaneously inhibiting both gatekeeper and solvent-front resistant mutants. Results of Western blotting showed that BYS10 dose-dependently degraded RET protein levels in the Ba/F3-KIF5B-RET-V804L xenograft model with immune deficiency and significantly inhibited the phosphorylation of RET kinase in tumor tissues. This study indicates that BYS10 exerts its anti-proliferative effect by blocking the RET signaling axis, although the specific downstream pathways require further investigation.

Preclinical evaluation revealed that BYS10 demonstrates potent RET-selective antitumor activity across both *in vitro* and *in vivo* models, effectively suppressing clinically critical resistance mutations in gatekeeper and solvent-front regions. Additionally, no significant changes in mouse body weight were observed in the BYS10-treated groups, indicating that BYS10 has good safety at the specified doses. Taken together, BYS10 is a potent and selective RET inhibitor, which has great potential to be translated into a clinically effective drug.

## Data Availability

The original contributions presented in the study are included in the article/Supplementary Material, further inquiries can be directed to the corresponding authors.
